# Different Responses of Invasive Weed *Alternanthera philoxeroides* and *Oryza sativa* to Plant Growth Regulators

**DOI:** 10.3390/life12071069

**Published:** 2022-07-17

**Authors:** Jiahao Wang, Die Hu, Xinning Shi, Jing Luo, Guangqian Ren, Zhicong Dai, Shanshan Qi, Daolin Du

**Affiliations:** 1 School of Agricultural Engineering, Jiangsu University, Zhenjiang 212013, China; 2212016005@stmail.ujs.edu.cn (J.W.); hu_209die@163.com (D.H.); 3192501013@stmail.ujs.edu.cn (J.L.); 2 School of the Environment and Safety Engineering, Jiangsu University, Zhenjiang 212013, China; sxn13753938033@163.com (X.S.); rgq@ujs.edu.cn (G.R.); daizhicong@163.com (Z.D.); ddl@ujs.edu.cn (D.D.)

**Keywords:** alligator weed, RICE, gibberellic acid, paclobutrazol, invasive weeds, agricultural ecosystem

## Abstract

Invasive plants cause a global loss of biodiversity, pose a major threat to the environment and economy, and also significantly affect agricultural production and food security. Plant growth regulators (PGRs) are widely used in agricultural production and might also affect invasive weeds distributed around crops in various ways. At present, there are few studies concerning whether there are significant effects of PGRs on invasive weeds. In this study, two widely used PGRs in paddy fields, gibberellic acid (GA) and paclobutrazol (PAC), were applied on *Oryza sativa* and a noxious weed *Alternanthera philoxeroides*, which is frequently distributed in paddy fields. The purpose of this study was to investigate if there are different responses of rice and weeds to these two plant regulators and the significant effects of PGRs on invasive weeds. The results showed that GA significantly promotes the total biomass of *A. philoxeroides* by 52.00%, but does not significantly affect that of *O. sativa*. GA significantly increases the growth of aboveground and belowground *A. philoxeroides*, but not that of *O. sativa*. On the other hand, PAC extremely inhibited the aboveground and belowground biomass of *A. philoxeroides* by more than 90%, but did not significantly inhibit the belowground biomass of *O. sativa*. PAC also enhanced the leaf nitrogen content and chlorophyll content of *A. philoxeroides*, but not the traits of *O. sativa*. Therefore, the effects of PGRs are significantly different between rice and the invasive weed. The potential promotion effects of PGRs on weeds that are frequently distributed in farmland warrant sufficient attention. This is probably one of the important reasons why invasive weeds can successfully invade the agricultural ecosystem with large human disturbance. This study might sound an alarm for weed control in paddy fields.

## 1. Introduction

The invasion of alien species leads to the loss of biodiversity, disrupts the stability of ecosystems, and poses a major threat to the environment and economy [1,2,3]. Invasive plant species lead to huge losses in global agriculture and adversely affect food security [4]. Invasive weeds are regarded as a major threat to global agriculture [5], and have many negative effects on crops, including competition with native plants for resources, rapid growth, and they could be host plants for pests and pathogens [6].

*Alternanthera philoxeroides* (alligator weed), native to South America, is a clonal weed in the family of Amaranthaceae and was introduced into China in the 1930s [7]. This species has become an invasive plant of global proportions, spreading to more than 30 countries [8]. *A. philoxeroides* has now invaded a variety of different ecosystems, including wetland ecosystems, lake ecosystems, and farmland ecosystems [9,10].

**Figure 1:**
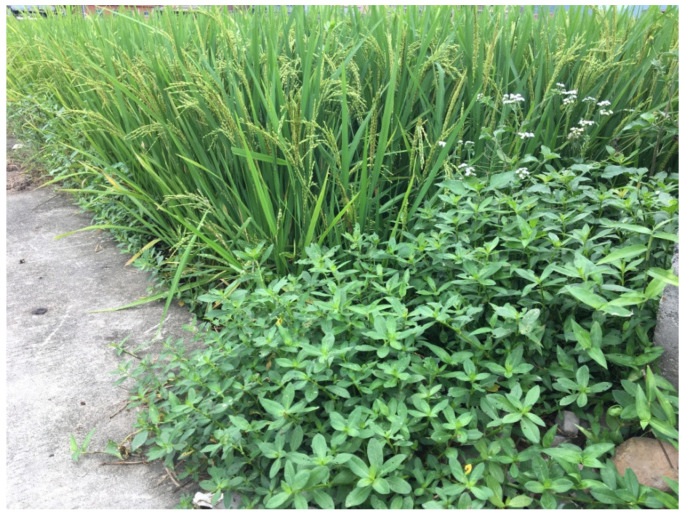
*Alternanthera philoxeroides* in a paddy field.

Rice (*Oryza sativa*) is the main crop and the staple food of an estimated 3.5 billion people all over the world. It is the most cultivated and renowned agricultural crop around the world [11]. More than 80% of rice is produced in Asia, and China is the world’s largest producer of rice [12]. However, it is reported that invasive weeds represent one of the most important factors affecting rice yield [13]. Alligator weed is also considered a harmful weed in China’s rice fields [14,15]. We found that *A. philoxeroides* is particularly common in rice paddies (Figure 1). Alligator weed has been reported to seriously reduce the yield of rice, maize, and vegetable crops [16,17,18]. Currently, most of the research on *A. philoxeroides* focuses on its growth characteristics and how to control and reduce its harmful effects on the environment. Ge et al. (2018) found that *A. philoxeroides* could inhibit the growth of native plants via allelopathic effects on soil enzyme activity and the microbial community [19].

Plant growth regulators (PGRs) are widely used in agricultural ecosystems and play an important role in improving crop yield [20]. Plant growth and development, insect resistance, and disease resistance are regulated by various PGRs [21]. Gibberellin (GA) plays an important role in the process of plant growth and development; it can promote seed germination, vegetative growth, and fruit development [22,23]. GA plays a key role in inhibiting leaf senescence, which can improve tomato yield by inhibiting leaf senescence in a stressful environment [24]. GA is a stimulant for deep-water *Oryza sativa,* which survives in water by promoting internode elongation [25]. However, for common *O. sativa* or wheat, GA may lead to excessive internode lodging and reduced yield. Tang et al. (2021) found that wheat with GA-insensitive genes achieved success in increasing yield [26]. Compared with GA, paclobutrazol (PAC), a GA biosynthesis inhibitor [27], has been widely used in rice fields because it can inhibit plant elongation and dwarf plants, thus increasing plant density and resistance to overwhelming, and it improves rice yield and quality [28,29]. When PGRs are applied to rice field crops, they may be directly sprayed on invasive weeds around crops or absorbed by invasive weeds from soil due to rainfall infiltration [30,31]. Therefore, the application of PGRs may also affect the growth of invasive weeds in farmland, which may cause serious harm to agricultural ecosystems. Previous studies have reported that fertilizer applied to crops might affect the invasion of weeds in farmland. Wan et al. (2012) found that long-term fertilization resulted in the growth of invasive weeds in fields and increased the biomass of invasive weeds [32]. However, the effects of PGRs on invasive weeds in farmland are not given enough attention. During the growing season, *A. philoxeroides* in paddy fields is often exposed to PGRs, such as GA and PAC. However, the effects of these PGRs on *A. philoxeroides* have not been fully studied. Considering the different effects of GA and PAC on plant growth, we investigated the response of *A. philoxeroides* and *O. sativa* to two identical concentrations of PGRs (GA and PAC). We addressed the following questions: (1) Do *A. philoxeroides* and *O. sativa* have different response abilities to these two PGRs? (2) Do these two PGRs significantly affect the growth of *A. philoxeroides*?

## 2. Materials and Methods

### 2.1. Experimental Materials

*A. philoxeroides* fragments were collected from a greenhouse of Jiangsu University, Zhenjiang, China. Seeds of japonica rice were purchased from a local seed company. Stem segments of *A. philoxeroides* with two nodes and 10 cm height seedlings of *O. sativa* germinated from seeds were selected for the experiment. The stem segments of *A. philoxeroides* and *O. sativa* seedlings were placed in plastic flowerpots (9 × 6 × 7.5 cm), which were filled with washed, sterilized, and dried river sand.

In order to study the effect of PGRs on the growth of *A. philoxeroides* and *O. sativa*, 50 mL of 30 μM GA and 30 μM PAC were applied to *A. philoxeroides* segments and *O. sativa* seedlings, respectively. The concentrations of GA and PAC were used as they are utilized in rice fields to increase crop yields [33] and prevent lodging [34] in paddy fields. The same volume of distilled water was added as a control treatment (CK). There were six treatments in this experiment: two plant species (*A. philoxeroides* and *O. sativa*) with three hormone treatments (CK, GA, PAC), and seven replications for each treatment. There were 21 *A. philoxeroides* stems and 21 *O. sativa* seedlings, with one stem or seedling per pot, planted in 42 plastic pots containing 100 g of sand and vermiculite (weight ratio: 2:1). All the plants were randomly arranged in the greenhouse and rotated once per week with natural light. Hoagland’s nutrient solution was added to seedlings every week to meet plant nutritional needs. In this study, PGRs were directly applied to the base of *A. philoxeroides* to simulate what might happen under natural conditions in which rain erosion and soil infiltration may expose PGRs to the base of plants.

### 2.2. Growth Trait Measurements

All the plants were harvested after two months of growth. Various growth indicators of the plants were measured, including plant height, root length, leaf nitrogen content, relative chlorophyll content, root area, root volume, aboveground dry mass, and belowground dry mass, and the total dry mass was calculated. The plant height and root length were measured with a ruler, the stem base of the plant was measured with a vernier caliper, and the relative chlorophyll content and leaf nitrogen content were measured with a SPAD-502 chlorophyll content analyzer. Root area and root volume were measured with the WinRHIZO root scanner system. The harvested plant material was dried in a constant temperature drying oven at 80 °C for 72 h, and the dry mass was determined.

### 2.3. Data Analysis

SPSS software was used for the statistical analysis of the data, and Tukey’s honest significant test (HSD) was used to compare the differences in the growth of *A. philoxeroides* and *O. sativa* among PGR treatments.

## 3. Results

### 3.1. Responses of Alternanthera philoxeroides and O. sativa to PGR

The results showed that PGRs have significant effects on most growth indicators of *A. philoxeroides* and *O. sativa* (Table 1).

**Figure 2:**
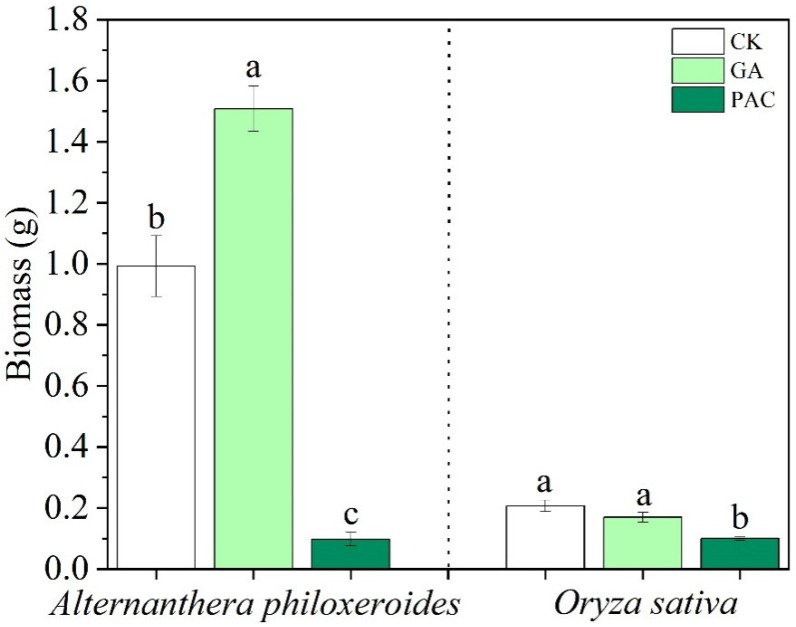
Effects of plant growth regulators (GA and PAC) on total biomass of *Alternanthera philoxeroides* and *Oryza sativa*. CK represents the control treatment. Bars show mean ± SE (*n* = 7). Different letters indicate significant differences (*p* < 0.05) among the three treatments of PGRs.

Compared with *O. sativa*, *A. philoxeroides* is more sensitive to these two PGRs. GA significantly promoted the biomass of *A. philoxeroides* by 52.00% but had no effects on the biomass of *O. sativa* (Figure 2). The application of PAC decreased the total biomass of *A. philoxeroides* by 90.13%, and also significantly decreased that of *O. sativa* by 51.38% (Figure 2).

### 3.2. Responses of Alternanthera philoxeroides and Oryza sativa to PGRs in Aboveground Growth

**Figure 3:**
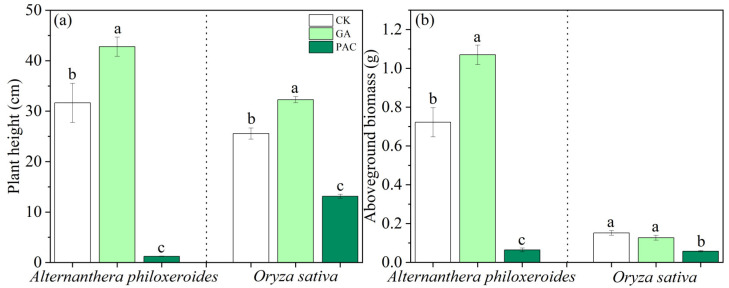
Effects of plant growth regulators (GA and PAC) on (**a**) plant height and (**b**) aboveground biomass of *Alternanthera philoxeroides* and *Oryza sativa*. CK represents the control treatment. Bars show mean ± SE (*n* = 7). Different letters indicate significant differences (*p* < 0.05) among the three treatments of PGRs.

The results showed that the application of GA significantly promoted the plant height of *A. philoxeroides* and *O. sativa*, while the application of PAC significantly inhibited the plant height of *A. philoxeroides* and *O. sativa* (Figure 3a). The same concentration of PAC extremely inhibited the plant height of *A. philoxeroides* by 96.16%, and inhibited that of *O. sativa* by 48.60%.

GA significantly increased the aboveground biomass of *A. philoxeroides* (Figure 3b). However, compared with the control treatment, the aboveground biomass of *O. sativa* was not significantly affected by GA application (Figure 3b). Meanwhile, PAC significantly inhibited the aboveground biomass of *A. philoxeroides* and *O. sativa*. However, PAC extremely inhibited the aboveground biomass of *A. philoxeroides* by 91.13%, and inhibited the aboveground biomass of *O. sativa* by 51.38%.

### 3.3. Responses of Alternanthera philoxeroides and Oryza sativa to PGRs in Belowground Growth

**Figure 4:**
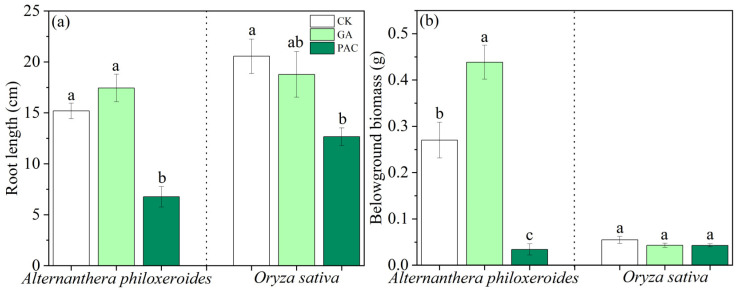
Effects of plant growth regulators (GA and PAC) on (**a**) root length and (**b**) belowground biomass of *Alternanthera philoxeroides* and *Oryza sativa*. CK represents the control treatment. Bars show mean ± SE (*n* = 7). Different letters indicate significant differences (*p* < 0.05) among the three treatments of PGRs.

GA slightly, but not significantly, increased the root length of *A. philoxeroides*, and it slightly inhibited the root length of *O. sativa*. PAC had a significant inhibitory effect on the root length of both plant species (Figure 4a).

The application of GA significantly promoted the belowground biomass of *A. philoxeroides*, and the PAC treatment significantly inhibited the belowground biomass of *A. philoxeroides*. However, both PGRs had no significant effects on the belowground biomass of *O. sativa* (Figure 4b).

**Figure 5:**
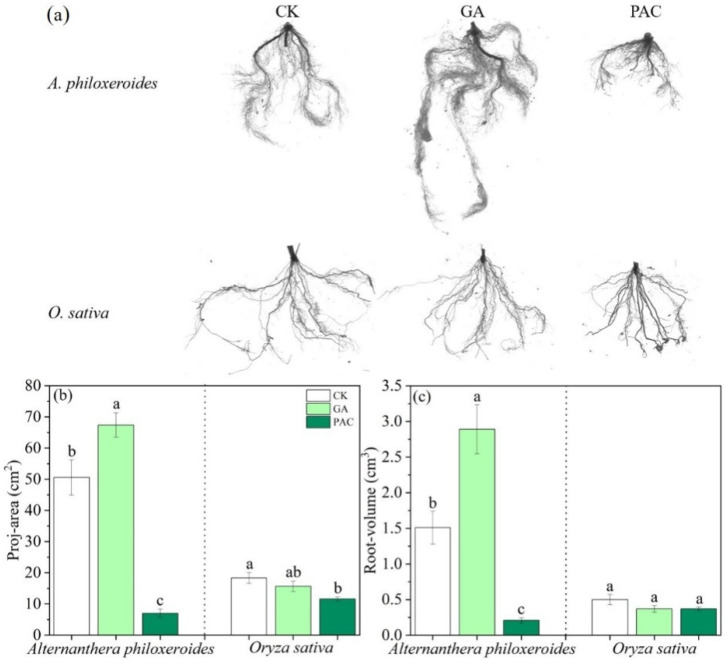
Effects of plant growth regulators (GA and PAC) on (**a**) root morphology (**b**) root area and (**c**) root volume of *Alternanthera philoxeroides* and *Oryza sativa*. CK represents the control treatment. Bars show mean ± SE (*n* = 7). Different letters indicate significant differences (*p* < 0.05) among the three treatments of PGRs.

For the morphology traits of roots, GA significantly increased the root area of *A. philoxeroides*, while GA had no significant effect on *O. sativa* (Figure 5a,b). PAC significantly reduced the root area of both two plant species (Figure 5a,b). Compared with the control treatment, GA significantly increased the root volume of *A. philoxeroides* and PAC significantly inhibited the root volume of *A. philoxeroides*. However, both PGRs had no significant effect on the root volume of *O. sativa* (Figure 5a,c).

### 3.4. Response of Alternanthera philoxeroides and Oryza sativa to PGRs in Physiological Traits

**Figure 6:**
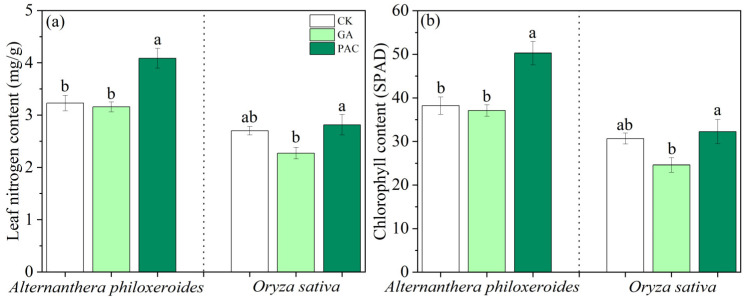
Effects of plant growth regulators (GA and PAC) on (**a**) leaf nitrogen content and (**b**) chlorophyll content of *Alternanthera philoxeroides* and *Oryza sativa*. CK represents the control treatment. Bars show mean ± SE (*n* = 7). Different letters indicate significant differences (*p* < 0.05) among the three treatments of PGRs.

In terms of physiological indicators, GA had no significant effect on leaf nitrogen content or relative chlorophyll content of *A. philoxeroides*, while PAC significantly increased these two physiological traits of *A. philoxeroides*. However, leaf nitrogen content and relative chlorophyll content of *O. sativa* were not significantly changed after the application of these two PGRs (Figure 6).

## 4. Discussion

The results of this study show that the responses of *A. philoxeroides* and *O. sativa* are quite different for the two PGRs, GA and PAC. Furthermore, these two PGRs have significant effects on the growth of *A. philoxeroides*.

PGRs are among the important components of agricultural production. They play important roles in promoting crop development and improving crop yields. They have been widely used in agriculture systems all over the world [35,36]. GA plays an important role in promoting plant growth and shoot elongation [37]. In this study, we found that GA significantly elongated both the invasive weed *A. philoxeroides* and the crop *O. sativa*. However, GA significantly enhanced both the biomass and the root morphology of the invasive weed, but not the biomass or root morphology of the rice crop. The growth of *A. philoxeroides* was more sensitive than rice to the same concentration of GA. Previous studies have found that GA has stronger effects on invasive plants [38], which is consistent with our results. From the physiology traits results, the addition of exogenous GA significantly inhibited leaf nitrogen content and chlorophyll content of *O. sativa*. It is reported that DELLAs, which negatively regulate gibberellin signaling to repress GA-mediated responses, positively regulate chlorophyll biosynthesis [39]. However, GA did not inhibit the chlorophyll content of *A. philoxeroides*. These might contribute to the photosynthesis of the invasive weed and promote its growth and development.

Our results showed that the effects of PAC, a synthetic inhibitor of GA, on the growth of *A. philoxeroides* and *O. sativa* are completely opposite to GA. PAC significantly inhibited shoot elongation and biomass of both plants. The addition of PAC to *A. philoxeroides* also significantly inhibited its belowground biomass. Dai et al. (2016) found that PAC inhibited the aboveground and belowground growth of an invasive plant, *Sphagneticola trilobata* [40], which is consistent with what we found. However, the belowground biomass of *O. sativa* treated with PAC did not change significantly. The effect of PAC on the growth of belowground also changed due to different species [41,42]. Consistent with this, the belowground growth of *A. philoxeroides* and *O. sativa* responds differently to PAC. In this case, although PAC inhibited the biomass of *A. philoxeroides*, the leaf nitrogen content and chlorophyll content of *A. philoxeroides* were significantly increased with PAC application. PAC induction in plants leads to the reduction in endogenous GA content [43,44,45]. Endogenous GA is extremely important when encountering biotic or abiotic stress. Endogenous GA reduction might increase flavonoid content in plants [46] and contribute to improving plant ability to resist stress [47]. Therefore, the chlorophyll and leaf nitrogen increasing due to PAC application might affect plant resistance to abiotic or biotic stress. Furthermore, some studies showed that PGRs increased the resistance of invasive weed *A. philoxeroides* to pathogenic bacteria and promoted its invasion [48]. Meanwhile, as a clonal plant [49], the clonal integration of *A. philoxeroides* improves its competitiveness [50]. Furthermore, there are other chemicals such as fertilizers applied in paddy fields affecting the growth of invasive weeds [51,52]. These chemicals might not only affect the growth but also the resistance to biotic or abiotic stresses of invasive weeds [53].

In the present study, we found that *A. philoxeroides* responded stronger to PGRs than *O. sativa*. According to present results, PGRs significantly affected the weed growth, and this might be one of the important factors contributing to the invasive weed’s rapid growth and spread in paddy fields. The effects of PGRs on invasive weeds should be seriously considered when planning for the prevention and control of invasive clonal species in agricultural and natural ecosystems.

## 5. Conclusions

In the present study, we confirmed that *A. philoxeroides* and *O. sativa* respond differently to PGRs, and PGRs significantly affected the growth of invasive weeds. Thus, the effects of PGRs on plant growth might be species-specific; more research is needed to provide further insights into PGRs, including different responding mechanisms to PGRs between invasive weeds and crops. The impacts of PGRs on invasive weeds have been rarely studied; however, the application of PGRs in farmland may lead to potential harmful effects on the agriculture system, including invasive weeds’ fast growth, which should be seriously considered. Thus, we appeal against the abuse of PGRs in paddy fields. On the other hand, since invasive weeds are more sensitive to PGRs than rice, genetic engineering, such as RNA interference technology, could be used to block plant hormone signaling pathways to control invasive weeds.

## Figures and Tables

**Table 1:** The effects of PGRs on the growth of *Alternanthera philoxeroides* and *Oryza sativa* (7 replications).

Species	Traits	df	F	*p*	Mse	Mst	Cov
*Alternanthera philoxeroides*	Plant height	2	74.048	<0.001	43.773	3241.286	75.606%
	Root length	2	27.774	<0.001	7.995	222.040	41.300%
	Aboveground biomass	2	93.935	<0.001	0.019	1.826	72.317%
	Belowground biomass	2	41.677	<0.001	0.007	0.289	75.742%
	Biomass	2	96.455	<0.001	0.037	3.563	72.071%
	Chlorophyll content	2	12.450	<0.001	29.997	373.453	19.162%
	Leaf nitrogen content	2	12.015	<0.001	0.156	1.869	16.380%
	Proj-area	2	58.984	<0.001	115.391	6806.280	67.265%
	Root volume	2	31.239	<0.001	0.402	12.556	83.137%
*Oryza sativa*	Plant height	2	161.890	<0.001	4.079	660.333	35.277%
	Root length	2	5.977	0.010	20.100	120.147	31.663%
	Aboveground biomass	2	22.586	<0.001	0.001	0.017	43.088%
	Belowground biomass	2	1.531	0.243	0.000	0.000	31.725%
	Biomass	2	13.285	<0.001	0.002	0.020	36.746%
	Chlorophyll content	2	4.091	0.034	28.028	114.652	20.768%
	Leaf nitrogen content	2	4.259	0.031	0.135	0.573	16.278%
	Proj-area	2	5.554	0.013	14.495	80.504	30.197%
	Root volume	2	2.328	0.126	0.019	0.043	35.366%

df = degrees of freedom, *p* = significance, Mse = mean of squares for error, Mst = mean of squares for treatment, Cov = coefficient of variation.

## Data Availability

The data presented in this study are available on request from the corresponding author (e-mail: qishanshan1986120@163.com).
